# Financing the future of TB control: from dependence to resilience

**DOI:** 10.5588/pha.25.0037

**Published:** 2025-12-03

**Authors:** G.N. Kazi

**Affiliations:** Editor in Chief, Public Health Action, The Union, Paris, France.

**Keywords:** tuberculosis, USAID, pandemic preparedness, global health security, funding gap

## Abstract

Recent cuts in international donor funding threaten global progress in TB control, particularly in countries that are heavily reliant on external support. With TB still the deadliest infectious disease, reduced funding could lead to millions of preventable cases and deaths. However, this crisis also presents an opportunity: governments must increase domestic investment, integrate TB care into broader health systems, and build resilient, patient-centered services. Doing so strengthens pandemic preparedness and addresses climate-related health risks. Ultimately, sustained progress against TB requires strong national leadership to move from donor dependency to self-reliant, equitable and sustainable health systems.

Recent reductions in international donor funding for health – and particularly TB control – threaten to reverse decades of progress.^1–3^ For many low- and middle-income countries, TB programs have relied heavily on external support to sustain essential services such as diagnostics, treatment, community outreach, and research.^4,5^ Multilateral and bilateral donors have long underpinned these systems, often providing the bulk of national TB budgets. With these contributions now substantially reduced, the sustainability of TB responses is in question. The timing could not be more critical: TB remains the world’s deadliest infectious disease, with 10.8 million new cases and 1.25 million deaths reported in 2023, including 161,000 among people living with HIV.^6^ The scale of the challenge is enormous. Modeling studies predict that without adequate funding, millions of additional TB cases and deaths could occur by 2035.^7^ The consequences extend well beyond mortality. TB is closely tied to poverty, and when patients face treatment costs without financial protection, families are often driven into catastrophic expenditures.^8,9^ This not only worsens inequality but also undermines the national economic progress and the race toward universal health coverage (UHC). Every year, TB pushes hundreds of thousands of households into economic crisis, creating a vicious cycle of ill health and impoverishment. Abrupt reductions in donor support risk dismantling decades of investment in laboratories, supply chains, community health worker networks, and patient support systems.

Yet amid these dangers lies an opportunity for transformation, as the crisis underscores the imperative for national governments to assume greater responsibility for financing their TB responses.^10^ Domestic funding offers clear benefits: it provides stability in the face of volatile donor flows, ensures accountability to citizens, and aligns resources more closely with local needs. Encouragingly, several countries are already moving in this direction. Nigeria has pledged increased national allocations for TB, the Democratic Republic of Congo has invested in local drug production to reduce reliance on imports, and South Africa has expanded treasury funding, embedding TB within broader national health plans.^10^ These examples illustrate that political will and financial commitment can begin to close the gap left by donors.

The necessary health reforms, however, need to transcend financing issues alone. For decades, TB has often been managed through vertical, disease-specific programs. While this approach enabled rapid scaling of services, it is increasingly inadequate. Patients do not experience TB in isolation; many also suffer from other chronic respiratory conditions, such as silicosis, chronic obstructive pulmonary disease, asthma, or post-TB lung damage. Treating these conditions separately wastes resources and often fails to meet the real needs of the patient. Integration of TB with broader lung health services and primary health care (PHC) systems enables a more patient-centered approach, improves efficiency and reduces duplication. The COVID-19 pandemic demonstrated the vulnerability of vertical programs, which were severely disrupted, while integrated systems were better able to adapt and continue serving patients.^11^ Embedding TB within holistic care with community involvement and multi-sectoral action is therefore a more effective, resilient and viable option.^12^ This integration also strengthens preparedness for the future. The TB infrastructure (laboratories, surveillance systems, and health workforce) forms part of the backbone of pandemic preparedness and global health security. Sustaining and expanding these systems enables countries to respond more effectively to emerging threats (from novel influenza viruses to coronaviruses). At the same time, climate change presents new challenges. Rising air pollution, population displacement, food insecurity, and changing environmental conditions all intersect with TB and other lung diseases, heightening vulnerability. Stable, integrated, and domestically funded health systems are therefore not only essential for TB control but also helpful in addressing the impacts of climate change and protecting populations against converging crises.

To realize this vision, financing reform is indispensable. Domestic investment in TB should be embedded within broader PHC budgets, ensuring that patients are protected from catastrophic costs. Rather than relying solely on donors, lines of collaboration may be cemented with organizations like The Union and other non-governmental organizations to reduce the funding gap by diversifying partnerships and reaching the maximum proportion of the population. The use of innovative and cost-effective diagnostics and medicines should also be explored. Additionally, governments could also develop innovative financing mechanisms such as social health insurance or expanding public–private partnerships to diversify resources. Importantly, greater reliance on domestic financing enhances governance: when taxpayers’ money is at stake, governments face stronger accountability and scrutiny. While international solidarity remains necessary – particularly given the cross-border nature of TB – the long-term trajectory must be one of partnership, where donor support complements, rather than substitutes, the national investment. Also, greater national spending will likely result in a better sense of ownership during the program design and implementation phases, leading to better outcomes.

Funding cuts are never welcome, especially when they threaten lives, but they can also serve as a wake-up call, highlighting the urgency of building resilient, self-reliant health systems to reduce uncertainty. By investing in integrated services that unite TB with broader lung health, national governments can turn this moment of uncertainty into an inflection point. They can safeguard progress against TB, advance UHC targets, and build systems better prepared for pandemics, global health security challenges, and the growing health impacts of climate change.^13,14^ However, one thing is certain, it is no longer business as usual and governments will need to realize that an investment in health will yield greater dividends in the future.

If national governments rise to this challenge, the current crisis could emerge as the catalyst for a healthier, more resilient and equitable future. TB control has long been framed as a global responsibility. It remains so, but increasingly, its success will depend on the strength of national leadership, the commitment of domestic resources, and healthcare that treats not only a single disease but provides an integrated approach.
